# Ginsenoside Rg2 Promotes the Proliferation and Stemness Maintenance of Porcine Mesenchymal Stem Cells through Autophagy Induction

**DOI:** 10.3390/foods12051075

**Published:** 2023-03-02

**Authors:** Lina Che, Caixia Zhu, Lei Huang, Hui Xu, Xinmiao Ma, Xuegang Luo, Hongpeng He, Tongcun Zhang, Nan Wang

**Affiliations:** 1 College of Biotechnology, Tianjin University of Science and Technology, Tianjin 300457, China; 2 Key Laboratory of Industrial Fermentation Microbiology, Ministry of Education, Tianjin 300457, China

**Keywords:** mesenchymal stem cells, cultivated meat, ginsenoside Rg2, proliferation, replicative senescence, autophagy

## Abstract

Mesenchymal stem cells (MSCs) can be used as a cell source for cultivated meat production due to their adipose differentiation potential, but MSCs lose their stemness and undergo replicative senescence during expansion in vitro. Autophagy is an important mechanism for senescent cells to remove toxic substances. However, the role of autophagy in the replicative senescence of MSCs is controversial. Here, we evaluated the changes in autophagy in porcine MSCs (pMSCs) during long-term culture in vitro and identified a natural phytochemical, ginsenoside Rg2, that could stimulate pMSC proliferation. First, some typical senescence characteristics were observed in aged pMSCs, including decreased EdU-positive cells, increased senescence-associated beta-galactosidase activity, declined stemness-associated marker *OCT4* expression, and enhanced P53 expression. Importantly, autophagic flux was impaired in aged pMSCs, suggesting deficient substrate clearance in aged pMSCs. Rg2 was found to promote the proliferation of pMSCs using MTT assay and EdU staining. In addition, Rg2 inhibited D-galactose-induced senescence and oxidative stress in pMSCs. Rg2 increased autophagic activity via the AMPK signaling pathway. Furthermore, long-term culture with Rg2 promoted the proliferation, inhibited the replicative senescence, and maintained the stemness of pMSCs. These results provide a potential strategy for porcine MSC expansion in vitro.

## 1. Introduction

With the growth of the world population and the economic development of developing countries, the demand for meat has increased rapidly [1,2,3]. It is estimated that by 2050, the global population will reach 9.5 billion [4]. To meet people’s demand for animal-based protein, the global meat production in 2050 is expected to increase to 169% of that in 2018 [5]. It is clear that traditional animal agriculture based on livestock and meat production methods cannot maintain the growth in the meat demand and will further exacerbate environmental stress. Recently, cultivated meat (CM), also known as in vitro meat, clean meat, cell-based meat, or cultured meat, was used as an alternative source of animal protein, providing a possible solution to these problems. In fact, meat is a set of complex muscle tissues, with a structure with specific characteristics and properties. Therefore, compared with the term “cultivated meat,” “food made with cultured animal cells” could describe this food more accurately at the current stage of development. Cultured animal cell food, as an important subfield of cellular agriculture, is produced in vitro using stem cells and tissue engineering, without sacrificing animals [6,7]. According to the ex ante life cycle assessment (LCA) of commercial-scale CM production in 2030 [8], compared to the traditional production of chicken, pork, and beef, it is estimated that industrialized CM production could reduce land use by 64%, 67%, and 55–90%, respectively. The carbon footprint of CM production is similar to that of chicken, which is significantly lower than that of pork and beef, and can be reduced by 43% and 67–92%, respectively. Food made with cultured animal cells is also beneficial to food security and animal welfare.

The primary types of cell sources for cultured animal cell food production mainly include pluripotent stem cells, such as embryonic stem cells (ESCs) and induced pluripotent stem cells (iPSCs), and adult stem cells, such as myosatellite cells and mesenchymal stem cells (MSCs) [6,9,10]. MSCs from a variety of animals, including chicken, pig, and bovines, have been shown to differentiate into adipocytes [11] and myocytes [12,13], thus producing fat and muscle, respectively. MSCs can be obtained mainly from bone marrow and also from other tissues, including adipose, umbilical cord, and placental tissues. Recent studies have focused on the use of MSCs for food made with cultured animal cells [14,15,16]. Zagury et al. constructed a three-dimensional fat-rich, edible engineered tissue by loading bovine MSCs within alginate hydrogel [14]. Machour et al. reported a “print-and-grow” approach using κ-carrageenan-based microgels (CarGrow), which was expected to be used in the production of CM [15]. MSCs printed and grown within CarGrow exhibited higher viability and proliferation capability compared to the control group. Hanga et al. developed a strategy for the expansion of bovine MSCs based on microcarriers [16]. Stem cell harvesting is the basis of cultured animal cell food production, and therefore, long-term culture and amplification of cells in vitro are required to obtain enough cells. However, the proliferative capacity of MSCs in vitro is limited. Long-term culture of MSCs in vitro leads to loss of stemness [17], and the cells undergo replicative senescence [18,19], which is accompanied by a decline in the differentiation potential [20], DNA damage response [19], anti-oxidation ability [21], and immune regulation ability [22,23]. Similar to MSCs derived from humans and mice, MSCs derived from porcine bone marrow or adipose tissue also suffer from replicative senescence after long-term in vitro passaging [11,23,24]. Therefore, it is of positive significance to study the biological characteristics of porcine MSCs’ (pMSCs) in vitro expansion and explore possible effective strategies to promote proliferation, maintain stemness, and inhibit replicative senescence.

Macroautophagy (hereafter referred to as autophagy) is a process that produces energy and macromolecular precursors for cellular renovation by degrading unnecessary or dysfunctional cell components, which is essential for maintaining cell, tissue, and organ homeostasis [25]. Activation of autophagy also helps to remove oxidized and damaged proteins and prevent the accumulation of toxic substances [26]. Aged MSCs are characterized by high levels of reactive oxygen species and accumulation of toxic or oxidized metabolites [27]. Recently, it has been reported that activation of autophagy can prevent radiation-induced ROS production and DNA damage in MSCs and therefore contributes to the preservation of stemness [28]. Additionally, blocking autophagy has been found to lead to ROS accumulation and stemness loss, suggesting that autophagy plays a crucial role in the maintenance of MSC stemness [28]. Similarly, Garcia-Prat et al. reported the important role of basal autophagy in preserving stemness in muscle satellite cells [29]. Compared to young quiescent satellite cells, autophagic activity in aged cells has been found to be impaired, while reactivation of autophagy could restore cellular stemness, rescue the proliferative defect, and reduce senescence [29]. However, the role of autophagy in the senescence of MSCs is still not fully understood, and results from the literature are controversial. For instance, autophagy has been reported to be activated in aged bone marrow MSCs (BMMSCs) due to the observed increase in autophagy-related gene expression [30]. Conversely, some recent investigations have stated that senescent BMMSCs have low or defective autophagy [31,32,33]. Therefore, it is imperative to explore the regulatory role of autophagy in porcine MSC stemness maintenance and senescence, which is critical for improving stem cell in vitro expansion.

Ginsenoside Rg2 is a biactive natural component of ginseng. The contents of Rg2 in the root of red ginseng (RG) are reported to be from 0.6 mg/g [34] to 1.1 mg/g [35]. Fermentation of ginseng with *Rhizopus oligosporus* increases the contents of Rg2 from 0.85 mg/g to 2.05 mg/g [36]. Black ginseng fermented with *Saccharomyces cerevisiae* contains 2.86 μg/mL of Rg2 [37]. Rg2 has been shown to have a variety of pharmacological effects, including anti-oxidant [38], anti-inflammatory [38], anti-cancer [39], cardiovascular protection [40], and neuro-protection [41,42] activities. Our previous study confirmed that Rg2 can activate autophagy in multiple mouse tissues and effectively improve cognitive impairment in mice with Alzheimer disease [41]. Recently, the other two ginsenosides, Rg1 [43] and Rg3 [44], were found to increase human BMMSC proliferation and suppress senescence in vitro. Moreover, it is reported that Rg1 is also able to improve the proliferative capacity of hematopoietic stem cells [45] and neural stem cells [46]. However, the effect of ginsenoside Rg2 on the proliferation and senescence of MSCs is unclear.

In this study, the senescence characteristics and autophagic activities of porcine MSCs during long-term culture in vitro were evaluated. Next, using a D-galactose (D-gal)-induced accelerated senescence model, we investigated the effect of ginsenoside Rg2 on the proliferation, senescence, and stemness of porcine MSCs and explored its potential mechanisms. Furthermore, whether Rg2 can stimulate the proliferation and maintain the stemness of porcine MSCs during long-term culture in vitro was also assessed.

## 2. Materials and Methods

### 2.1. Experimental Animals

In this study, 1–3-day-old pigs were obtained from the Tianjin Fushengyuan livestock farm. Animal experiments were performed in accordance with the guidelines established by the Institutional Animal Care and Use Committee at Tianjin University of Science & Technology.

### 2.2. Isolation and Culture of pMSCs

pMSCs were isolated from the femur and tibia of pigs according to the reported method with minor modifications [47]. Briefly, the femur and tibia were retrieved and rinsed twice with phosphate-buffered saline (PBS) containing 3% penicillin/streptomycin. After both ends of the femur and tibia were cut, the marrow was flushed out by inserting a syringe needle into the cut surface and centrifuged for 5 min at 1000 rpm at room temperature. The cells were resuspended in Dulbecco’s modified Eagle’s medium/F12 (DMEM/F12; Gibco, New York, NY, USA) containing 10% fetal bovine serum (FBS; AusGeneX, Gold Coast, Australia) and cultured at 37 °C in a humidified atmosphere containing 5% CO_2_. Porcine MSCs were passaged with digestion with 0.25% trypsin containing 0.02% EDTA when they reached 80–90% confluence. Cellular morphology was observed and photographed using a phase-contrast microscope (Nikon Eclipse Ti, Nikon, Tokyo, Japan).

### 2.3. Flow Cytometry

To identify cellular surface immunophenotypes, porcine MSCs were digested and washed twice with PBS. The cells were labeled with antibodies against PerCP-CD45 (Cat.#: 642275; BD Biosciences, New York, NY, USA), APC-CD44 (Cat.#: 103011; BioLegend, San Diego, CA, USA), FITC-CD90 (Cat.#: 328107; BioLegend, CA, USA), and PE-CD34 (Cat.#: 343605; BioLegend, CA, USA) for 30 min. After washing twice with PBS, the labeled cells were analyzed using a flow cytometer (BD Biosciences, New York, NY, USA).

To measure the intracellular reactive oxygen species (ROS) level, cells were incubated with 10 μM of DCFH-DA (Nanjing Jiancheng Biotechnology, Nanjing, China) at 37 °C for 1 h, followed by washing and resuspending with PBS. Fluorescence was analyzed via flow cytometry (BD Biosciences, New York, NY, USA) with excitation at 500 nm and emission at 525 nm.

### 2.4. Adipogenic and Osteogenic Differentiation of MSCs

For adipogenic differentiation, porcine MSCs (1 × 10^5^/well) at P4 were seeded in 6-well plates until they reached 70%–80% confluence. These cells were first cultured in adipogenic induction medium for 3 days and sequentially in maintenance medium for another 3 days. Next, the two media were replaced alternately until 21 days. Adipogenic induction medium is composed of DMEM/F-12 supplemented with 10% FBS, 10 μM of dexamethasone (Solarbio, Beijing, China), 200 μM of indomethacin (Solarbio, Beijing, China), and 10 μM of insulin (Solarbio, Beijing, China), while maintenance medium is composed of basal medium supplemented with 0.2 nM of insulin. After 21 days, the cells were fixed with 4% paraformaldehyde and then stained with oil red O (Solarbio, Beijing, China).

For osteogenic differentiation, porcine MSCs (1 × 10^5^/well) at P4 were seeded in 6-well plates until they reached 80–90% confluence. The medium was replaced with osteogenic induction medium. Osteogenic induction medium is composed of basal medium supplemented with 0.1 μM of dexamethasone (Solarbio, Beijing, China), 10 μM of β-glycerophosphate (Coolaber, Beijing, China), and 50 μM of vitamin C (Solarbio, Beijing, China). The media were changed every 2–3 days. After 21 days, the cells were fixed with 4% paraformaldehyde and then stained with alizarin red S (Solarbio, Beijing, China). 

### 2.5. Senescence-Associated β-Galactosidase (SA-β-Gal) Staining

To evaluate cellular senescence, β-gal activity was analyzed using a SA-β-Gal staining kit (Biyuntian, Beijing, China), following the manufacturer’s instructions. Briefly, pMSCs at P5, P10, and P15 were plated in a 6-well plate, fixed with fixative solution for 15 min at room temperature, and washed three times with PBS. The cells were incubated overnight with freshly prepared staining solution at 37 °C in the absence of CO_2_. After washing with 70% ethanol, the aging cells were dyed blue. The number of these blue cells was counted under a inverted phase-contrast microscope (Nikon, Tokyo, Japan).

### 2.6. Cell Proliferation Assay

Cellular proliferation was detected according to the instructions of a Click-iT EdU (5-Ethynyl-2′-deoxyuridine) Cell Proliferation Kit (Meilunbio, Dalian, China). pMSCs at P5, P10, and P15 were plated in a 24-well plate and cultured overnight. For labeling cells with EdU, an equal volume of 2× EdU solution was added to the cells, and the cells were incubated at 37 °C for 2 h. The samples were then fixed and permeabilized. The nuclei were stained using the Hoechst 33342 (Meilunbio, Dalian, China) fluorescent stain. Digital images were acquired using a laser confocal microscope (OLYMPUS, Tokyo, Japan), and the number of EdU-positive cells were calculated using Image-Pro Plus 5.1 software (MEDIA CYBERNETICS, Silver Spring, MD, USA). EdU incorporation (the ratio of EdU-labeled cells to total cells) indicated the cellular proliferation rate.

### 2.7. Quantitative Real-Time PCR (qRT-PCR)

Total RNA was isolated from porcine MSCs using Trizol reagent (Invitrogen, Carlsbad, CA, USA), and reverse transcription of the RNA sample to cDNA was carried out using M-MLV reverse transcriptase (Promega, Madison, WI, USA). qRT-PCR was performed on a Applied Biosystems StepOneTM RT-PCR system (Applied Biosystems, Foster City, CA, USA) with the Fast SYBR1 Green Master Mix obtained from Applied Biosystems. Primers for each targeted mRNA were designed and are listed in Table 1. The 2^−ΔΔCt^ method was used to calculate the relative expression levels of target genes, and *GAPDH* was used as an internal control.

### 2.8. Drug Administration

To monitor autophagic flux, pMSCs at P5, P10, and P15 were treated with 150 nM of bafilomycin A1 (Santa Cruz Biotechnology, Santa Cruz, CA, USA) for 2 h, and then, the protein samples were collected for LC3II detection. To assess the effect of Rg2 on cellular proliferation, Rg2 (Shanghai Yuanye Bio-Technology Co., Shanghai, China) was dissolved in DMSO and provided to the cells. pMSCs were treated with 25 μM, 50 μM, and 100 μM of Rg2 in DMEM/F-12 containing 1%, 5%, and 10% FBS for 24, 48, and 72 h, followed by MTT and EdU staining assays. To investigate the effect of Rg2 on D-gal-induced senescence, pMSCs were pre-treated for 24 h with 20 g/L of D-gal and then incubated with 100 μM of Rg2 in the presence/absence of D-gal for another 24 h, followed by MTT, EdU staining, SA-β-gal activity, and Western blot assays.

### 2.9. Western Blot

pMSCs treated under different conditions were collected and then lysed with RIPA buffer along with PMSF protease inhibitor. The primary antibodies used for immunodetection included anti-*OCT4* (Cat.#: AF0226; Affinity Biosciences, Changzhou, China), anti-p53 (Cat.#: AF0879; Affinity Biosciences, Changzhou, China; Cat.#: 10442-1-AP; Proteintech, Wuhan, China), anti-p62 (Cat.#: ab109012; Abcam, Cambridge, MA, USA), anti-LC3-I/II (Cat.#: NB100-2220; Novusbio, CO, USA), anti-p-AMPK (Cat.#: AF3423; Affinity Biosciences, Changzhou, China), anti-AMPK (Cat.#: sc74461; Santa Cruz Biotechnology, Santa Cruz, CA, USA), and anti-β-actin (Cat.#: sc8432; Signalway Antibody, Baltimore, MD, USA). The specific protein bands were visualized with the Odyssey Infrared Imaging System (LI-COR, Lincoln, Dearborn, MI, USA). The band density was analyzed using Image-Pro Plus 5.1 software (MEDIA CYBERNETICS, Silver Spring, MD, USA) using β-actin as an internal control and then normalized to the vehicle control.

### 2.10. Cell Viability Assay

For the detection of cellular viability, pMSCs (5 × 10^3^/well) were seeded in a 96-well plate with 100 μL of the medium, followed by MTT assay. The cells were treated with 5 mg/mL of 3-(4,5-dimethyl-thiazol-2-yl)-2,5-diphenyltetrazolium (MTT; Solarbio, Beijing, China) solution (10 μL per well) and then incubated for 4 h. The medium was then discarded, and 100 μL of dimethyl sulfoxide (DMSO) was added to each well. The absorbance of each well was measured using a Synergy 4 plate reader (Bioteck, Winooski, VT, USA) with a wavelength of 490 nm. Absorbance was directly proportional to the number of surviving cells.

### 2.11. Measurement of Malondialdehyde (MDA) Contents and Superoxide Dismutase (SOD) Activities

After pre-incubation with 20 g/L of D-gal for 24 h, pMSCs were incubated with 100 μM of Rg2 in the presence/absence of D-gal for another 24 h, and then, the cells were collected and lysed. The contents of MDA and the activities of SOD were detected using commercial available kits (Solarbio, Beijing, China) according to the manufacturer’s instructions.

### 2.12. Statistical Analysis

All data are shown as the mean ± SD, and all experiments were repeated at least three times. Statistical analysis was conducted using Microsoft Excel and GraphPad Prism 6. Two-tailed, unpaired Student’s *t*-tests were performed to determine statistical significance when comparing two groups, and one-way ANOVA followed by a Dunnett multiple-comparison test was used when comparing more than two groups. A *p*-value of <0.05 was considered statistically significant.

## 3. Results

### 3.1. Isolation, Culture, and Identification of pMSCs

**Figure 1:**
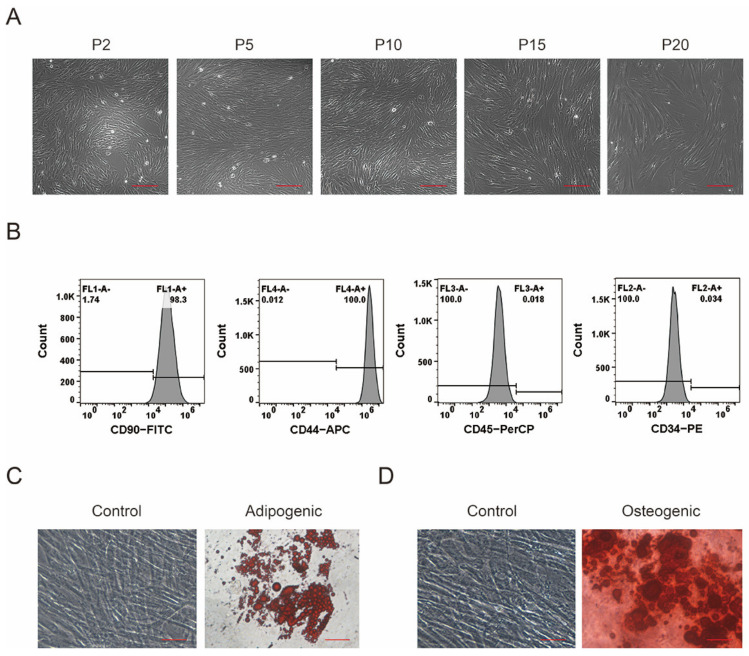
Isolation, culture, and identification of pMSCs. (**A**) Representative images of porcine MSCs at P2, P5, P10, P15, and P20. Scale bar = 200 μm. (**B**) Immunophenotypic analysis of pMSCs at P3 using flow cytometry. (**C**) Identification of adipogenic differentiation using oil red O staining. (**D**) Identification of osteogenic differentiation using alizarin red staining. Scale bar = 10 μm.

The primary cells isolated from porcine bone marrow were cultured in basic medium for 12 h and adhered to the wall. After 3–5 days, the cells began to fuse, and the rate of cell fusion reached 65%–70% within 1 week. As shown in Figure 1A, porcine MSCs at P2 and P5 showed a spindle shape and strong proliferative capacity, while the cells after several passages gradually showed the characteristics of aging, such as a flat body, hypertrophy, and weak refraction. Almost all cells lost their ability of proliferation beyond passage 20. To identify the immunophenotypes of the primary cells isolated, cellular surface markers CD34, CD44, CD45, and CD90 were analyzed in the cells at P3 using flow cytometry. The isolated porcine MSCs were strongly positive for CD44 (96.27 ± 0.13%) and CD90 (98.79 ± 0.05%) but negative for the hematopoietic lineage markers CD34 (0.08 ± 0.02%) and CD45 (0.04 ± 0.01%); see Figure 1B. In addition, the multi-lineage differentiation ability of MSCs to adipocytes and osteoblasts was studied. Lipid droplets and positive oil red O staining were observed in the pMSCs exposed to adipogenic differentiation medium for 21 days (Figure 1C), while calcified nodules and positive alizarin red S staining appeared in the cells exposed to osteogenic differentiation medium (Figure 1D). These results indicated that the primary cells isolated from porcine bone marrow were mesenchymal stem cells.

### 3.2. Reduced Proliferation Potential and Stemness in pMSCs after Long-Time Culture

**Figure 2:**
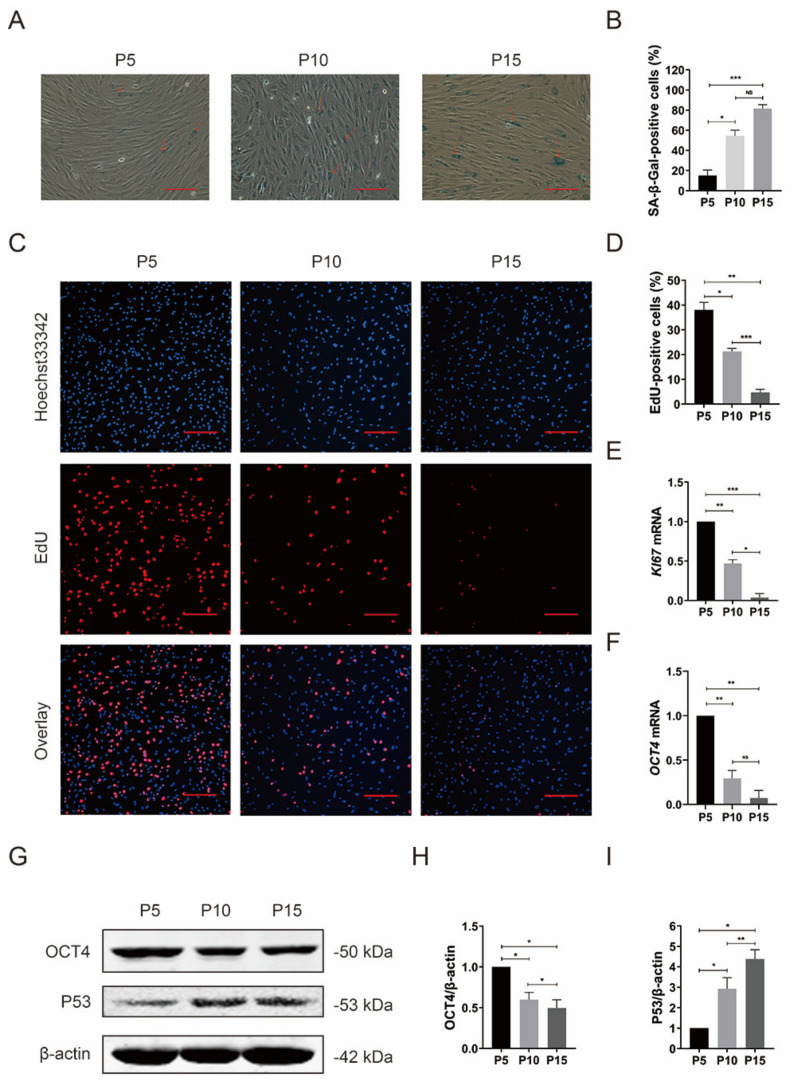
Reduced proliferation potential and stemness of pMSCs after long-time culture. (**A**) SA–β–gal staining of pMSCs at P5, P10, and P15. SA–β–gal–positive cells were indicated by red arrows. Scale bar = 10 μm. (**B**) Quantification of the SA–β–gal staining in (**A**). (**C**) EdU staining of pMSCs at P5, P10, and P15. Scale bar = 200 μm. (**D**) Quantification of the EdU staining in (**C**). (**E**) qRT–PCR analysis of *KI67* in pMSCs at P5, P10, and P15. (**F**) qRT–PCR analysis of *OCT4* in pMSCs at P5, P10, and P15. (**G**) Western blot analysis with anti–*OCT4* and anti-P53 antibodies in pMSCs at P5, P10, and P15. (**H**) Quantification of *OCT4* expression levels in (**G**). (**I**) Quantification of P53 expression levels in (**G**). * *p* < 0.05, ** *p* < 0.01, and *** *p* < 0.001; *n* = 3.

It is known that MSCs show reduced proliferation capacity and undergo replicative senescence with cellular expansion in vitro [48]. Here, some age-related changes were observed in pMSCs at P10 and P15 compared to the cells at P5. With an increase in the number of passages, the proportion of SA-β-gal-staining-positive cells significantly scaled up (Figure 2A,B), yet the proportion of EdU-positive cells decreased notably (Figure 2C,D). Next, we detected the mRNA levels of the stemness gene *OCT4* and the proliferative marker *Ki67* in pMSCs at P5, P10, and P15. The mRNA levels of *OCT4* and *Ki67* significantly decreased in pMSCs at higher passage numbers (Figure 2E,F). Consistent with the change in the *OCT4* mRNA level, the protein level of *OCT4* was downregulated in pMSCs at higher passage numbers (Figure 2G,H and Figure S1). Furthermore, a prominent increase in the protein level of the aging-related marker p53 was observed in pMSCs at P10 and P15 compared to the counterparts at P5 (Figure 2G,I and Figure S1).

### 3.3. Impaired Autophagic Flux and Elevated ROS in pMSCs after Long-Time Culture

**Figure 3:**
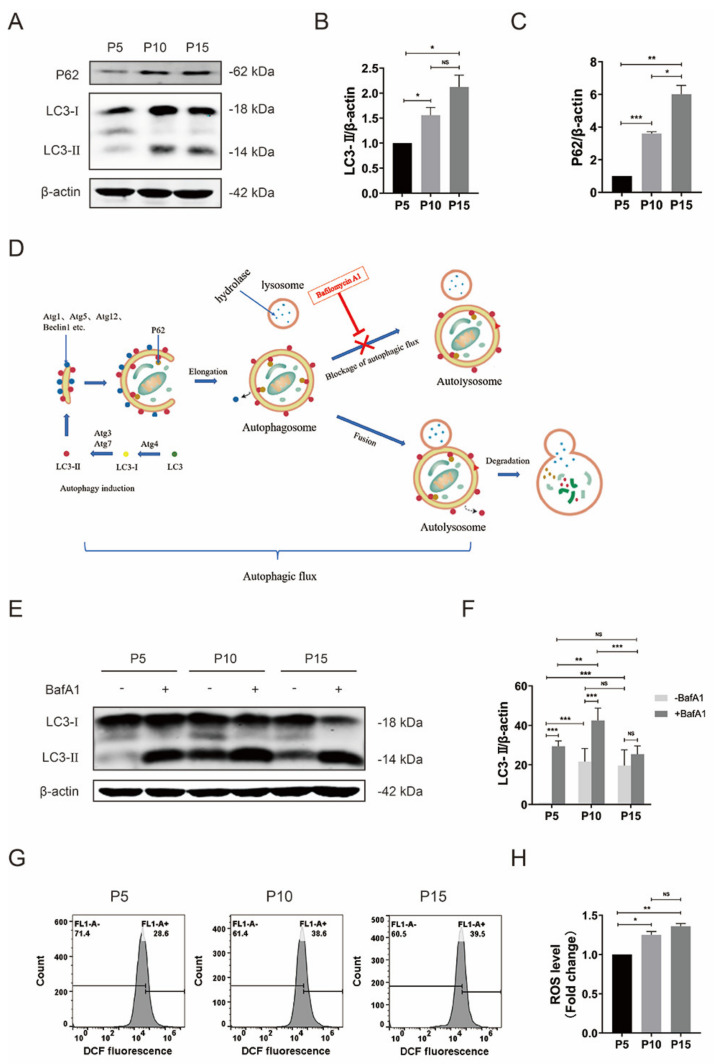
Impaired autophagic flux and elevated ROS in pMSCs after long-time culture. (**A**) Western blot analysis with anti–P62 and anti–LC3 antibodies in pMSCs at P5, P10, and P15. (**B**) Quantification of LC3−II expression levels in (**A**). (**C**) Quantification of P62 expression levels in (**A**). (**D**) Pattern diagram of autophagy. (**E**) Western blot analysis with anti–LC3 antibody in pMSCs after 2 h of BafA1 treatment. (**F**) Quantification of LC3−II expression levels in (**D**). (**G**) Histograms of ROS levels in pMSCs at P5, P10, and P15 using flow cytometry. (**H**) Quantification of intracellular ROS levels in (**F**). * *p* < 0.05, ** *p* < 0.01, and *** *p* < 0.001; *n* = 3.

The relationship between MSC senescence and autophagy remains unclear and debatable [26]. To investigate the relationship between the autophagy and replicative senescence of pMSCs, the expression of microtubule-associated protein 1 light chain 3 (LC3) and cargo protein SQSTM1/p62 was tested in pMSCs at P5, P10, and P15 using Western blot. As shown in Figure 3A–C and Figure S2, the relative protein levels of LC3-II and P62 in pBMSC significantly increased in aged pMSCs (P10 and P15) compared to young cells (P5). The increase in LC3-II indicates the combined results of increased autophagosome synthesis (activated autophagy induction) or suppressed autophagosome degradation (blockage of autophagic flux), while the accumulation of P62 indicates suppressed autophagic flux. To further distinguish between these two possibilities, BafA1 was used to block autophagosome–lysosome fusion (Figure 3D). Treatment with BafA1 for 2 h resulted in a noticeable accumulation of LC3-II in young pMSCs at P5, suggesting activated autophagic flux. Compared with BafA1-treated cells at P5, BafA1-treated pMSCs at P10 exhibited a further increase in LC3-II levels (Figure 3E,F and Figure S2), suggesting that in the early stages of aging, pMSCs can promote autophagy induction to remove toxic substrates. Although increased autophagosome synthesis was observed in pMSCs at P10, the accumulation of P62 in these cells (Figure 3A and Figure S2) suggested a defect in the later stages of autophagy (a potential inhibition of autophagosome degradation). Combined with markedly enhanced P62 levels, these results illustrate that senescent pMSCs activate autophagy at an early stage in response to oxidative stress, but the weakened autophagic flux makes it insufficient for them to completely remove toxic substances. Importantly, there was no significant difference in LC3-II levels between P5 and P15 pMSCs along with BafA1, but a profound increase in LC3-II levels was observed in P15 pMSCs without BafA1, compared to P5 cells, indicating that in the late stages of aging, autophagic flux is further impaired in pMSCs (Figure 3E,F and Figure S2).

Oxidative stress can cause oxidative damage to organelles and proteins, leading to cell senescence [49]. Correspondingly, we detected the ROS levels in pMSCs at P5, P10, and P15 using flow cytometry. As shown in Figure 3G,H, compared with young cells at P5, aged pMSCs at P10 and P15 showed a marked increase in ROS levels. These results further indicate the attenuated ability of senescent cells to scavenge ROS.

### 3.4. Ginsenoside Rg2 Promoted the Proliferation of pMSCs

**Figure 4:**
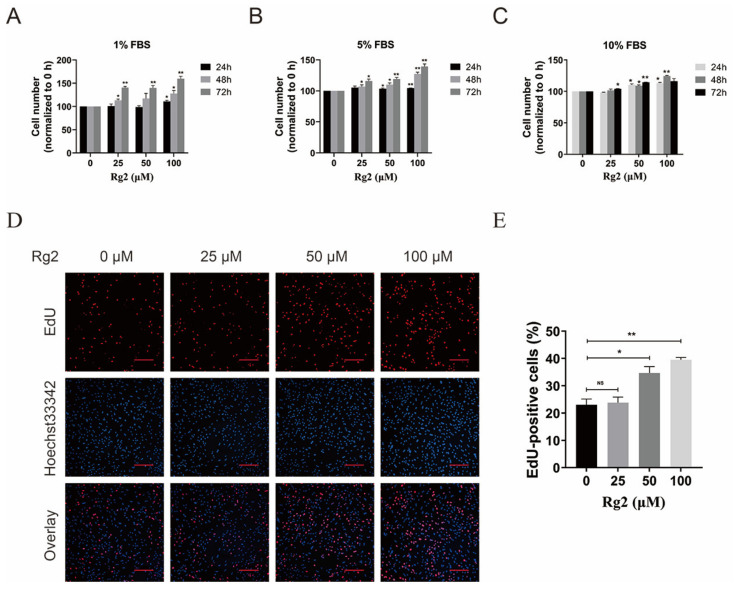
Ginsenoside Rg2 promoted the proliferation of pMSCs. (**A**) MTT assay of pMSCs after treatment with different concentrations of Rg2 in basic medium containing 1% FBS. (**B**) MTT assay of pMSCs after treatment with different concentrations of Rg2 in basic medium containing 5% FBS. (**C**) MTT assay of pMSCs after treatment with different concentrations of Rg2 in basic medium containing 10% FBS. (**D**) EdU staining of pMSCs after treatment with different concentrations of Rg2. Scale bar = 200 μm. (**E**) Quantification of the EdU staining in (**D**). * *p* < 0.05 and ** *p* < 0.01; *n* = 3.

To evaluate the stimulatory effect of ginsenoside Rg2 on the proliferation of porcine MSCs, cells at P6 were treated with different concentrations of Rg2 (25, 50, and 100 μM) in DMEM/F12 containing 1%, 5%, and 10% FBS for 24 h, 48 h, and 72 h, and then, MTT assay was carried out. Our data showed that ginsenoside Rg2 at a concentration of 25–100 μM exhibits no cytotoxicity and that cellular viability increased remarkably with increasing Rg2 concentration (Figure 4A–C). In addition, 100 μM of Rg2 showed the most significant proliferative effect under the condition of 1% serum (Figure 4A). Furthermore, the number of EdU-staining-positive cells markedly increased in pMSCs treated with 50 and 100 μM of Rg2 (Figure 4D,E). Our data showed that Rg2 can promote the proliferation of pMSCs in a concentration- and time-dependent manner.

### 3.5. Ginsenoside Rg2 Reversed D-Gal-Induced Senescence and Maintained Stemness in pMSCs

**Figure 5:**
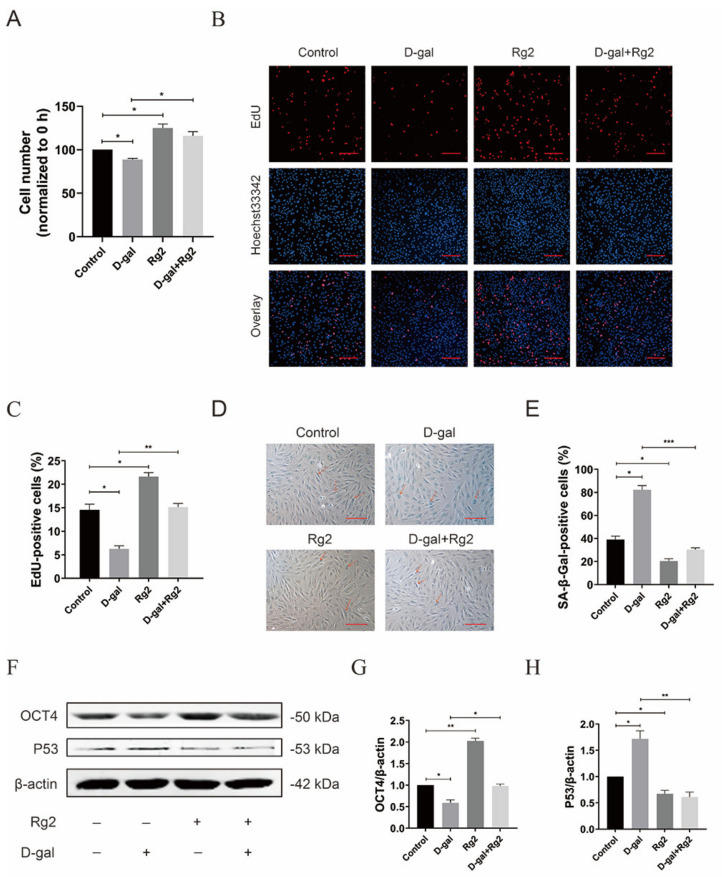
Ginsenoside Rg2 reversed D–gal–induced senescence and maintained stemness in pMSCs. After pre–incubation with 20 g/L of D–gal for 24 h, pMSCs were treated with 100 μM of Rg2 in the presence/absence of D–gal for another 24 h. (**A**) MTT assay of pMSCs after different treatments. (**B**) EdU staining of pMSCs after different treatments. Scale bar = 200 μm. (**C**) Quantification of the EdU staining in (**B**). (**D**) SA–β–gal staining of pMSCs after different treatments. SA–β–gal–positive cells were indicated by red arrows. Scale bar = 10 μm. (**E**) Quantification of the SA–β–gal staining in (**D**). (**F**) Western blot analysis with anti–*OCT4* and anti–P53 antibodies in pMSCs after different treatments. (**G**) Quantification of *OCT4* expression levels in (**F**). (**H**) Quantification of P53 expression levels in (**F**). * *p* < 0.05, ** *p* < 0.01, and *** *p* < 0.001; *n* = 3.

Next, the anti-senescence effect of Rg2 was assessed using a model of accelerated aging induced by D-gal. After pre-incubation with 20 g/L of D-gal for 24 h, pMSCs were treated with 100 μM of Rg2 in the presence/absence of D-gal for another 24 h and subsequently subjected to MTT, EdU staining, and SA-β-gal staining assays. Consistent with a previous study [50], we found that D-gal treatment significantly inhibited cell viability (Figure 5A), reduced the numbers of EdU-positive cells (Figure 5D,E) and increased the percentage of SA-β-gal-positive cells (Figure 5B,C). These changes mediated by D-gal were reversed by the administration of 100 μM of Rg2 (Figure 5A–E). Moreover, decreased *OCT4* levels induced by D-gal were rescued by the addition of Rg2 (Figure 5F,G and Figure S3), suggesting that Rg2 can contribute to the maintenance of pMSC stemness. Similarly, Rg2 significantly inhibited the D-gal-caused increase in the protein expression of P53 in pMSCs (Figure 5F,H and Figure S3). These results indicated that treatment with Rg2 effectively prevents the pro-senescence effects of D-gal on pMSCs.

### 3.6. Ginsenoside Rg2 Protected pMSCs against Oxidative Stress

**Figure 6:**
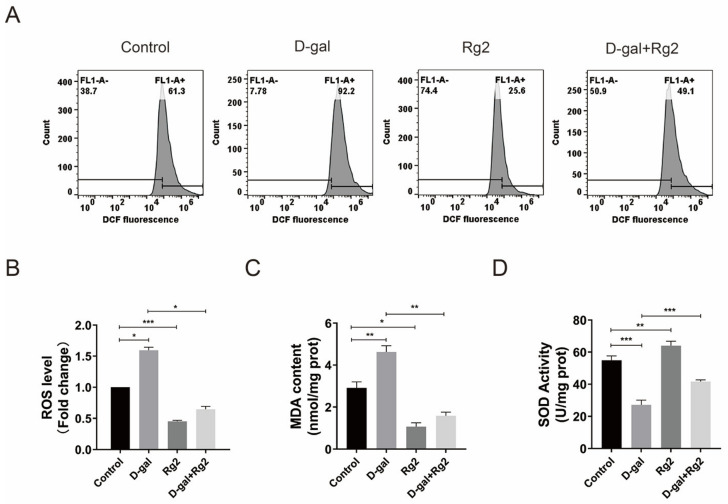
Ginsenoside Rg2 protected pMSCs against oxidative stress. After pre–incubation with 20 g/L of D–gal for 24 h, pMSCs were treated with 100 μM of Rg2 in the presence/absence of D–gal for another 24 h. (**A**) Histograms of ROS levels in pMSCs after different treatments using flow cytometry. (**B**) Quantification of intracellular ROS levels in (**B**). (**C**) MDA contents in pMSCs after different treatments. (**D**) SOD activities in pMSCs after different treatments. * *p* < 0.05, ** *p* < 0.01, and *** *p* < 0.001; *n* = 3.

To determine whether Rg2 can delay the senescence of pMSCs by reducing ROS levels, intracellular ROS levels were assessed in Rg2-treated pMSCs using flow cytometry. D-gal treatment markedly stimulated the production of ROS in pMSCs, whereas the effect was attenuated by the administration of Rg2 (Figure 6A,B). Furthermore, MDA contents and SOD activities were detected in Rg2-treated pMSCs. The addition of Rg2 dramatically inhibited the D-gal-stimulated increase in MDA contents (Figure 6C). SOD activities were significantly downregulated in D-gal-stimulated pMSCs, while Rg2 treatment partly reversed the D-gal-induced reduction in SOD activities (Figure 6D). These results indicated that Rg2 prevents the senescence of pMSCs by increasing SOD activities and reducing ROS and MDA levels.

### 3.7. Ginsenoside Rg2 Induced Autophagy in pMSCs via the AMPK Signaling Pathway

**Figure 7:**
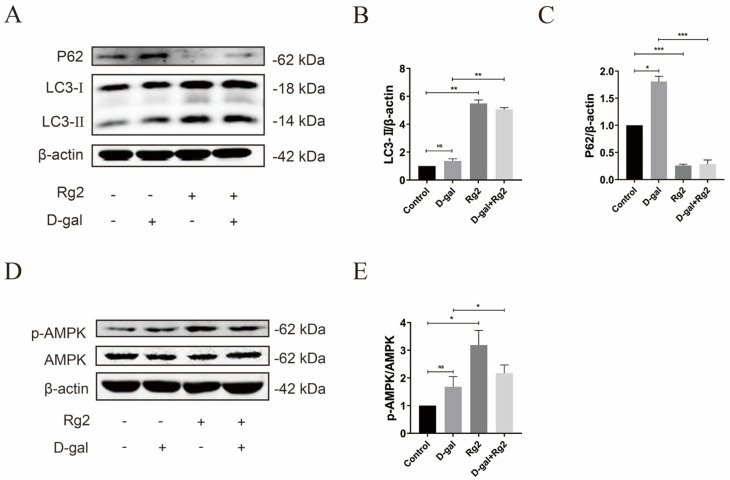
Ginsenoside Rg2 induced autophagy in pMSCs via the AMPK signaling pathway. After pre-incubation with 20 g/L of D–gal for 24 h, pMSCs were treated with 100 μM of Rg2 in the presence/absence of D–gal for another 24 h. (**A**) Western blot analysis with anti–P62 and anti–LC3 antibodies in pMSCs after different treatments. (**B**) Quantification of LC3 expression levels in (**A**). (**C**) Quantification of P62 expression levels in (**A**). (**D**) Western blot analysis with anti–p–AMPK and anti–AMPK antibodies in pMSCs after different treatments. (**E**) Quantification of p–AMPK/AMPK in (**D**). * *p* < 0.05, ** *p* < 0.01, and *** *p* < 0.001; *n* = 3.

To demonstrate whether the positive effect of Rg2 on the anti-senescence and stemness maintenance of pMSCs is related to autophagy induction, the protein expression of LC3 and P62 was tested in Rg2-treated pMSCs with/without D-gal using Western blot. Compared with the D-gal group, Rg2-treated cells showed increased LC3II expression and reduced P62 levels, indicating the activation of autophagy (Figure 7A–C and Figure S4). Our previous study confirmed that Rg2 can activate autophagy in multiple types of cells via the AMPK signaling pathway [41], but it is unknown whether Rg2 can activate the AMPK signaling pathway in porcine MSCs. Thus, we detected the expression of p-AMPK and AMPK in Rg2-treated pMSCs with/without D-gal using Western blot. As shown in Figure 7D,E and Figure S4, the relative protein level of p-AMPK/AMPK significantly increased in the Rg2 group compared to the D-gal group. These results further confirmed that autophagy activated by Rg2 can play a critical role in the anti-senescence and stemness maintenance of pMSCs via the AMPK signaling pathway.

### 3.8. Ginsenoside Rg2 Improved Longevity of pMSCs during Long-Term Culture

**Figure 8:**
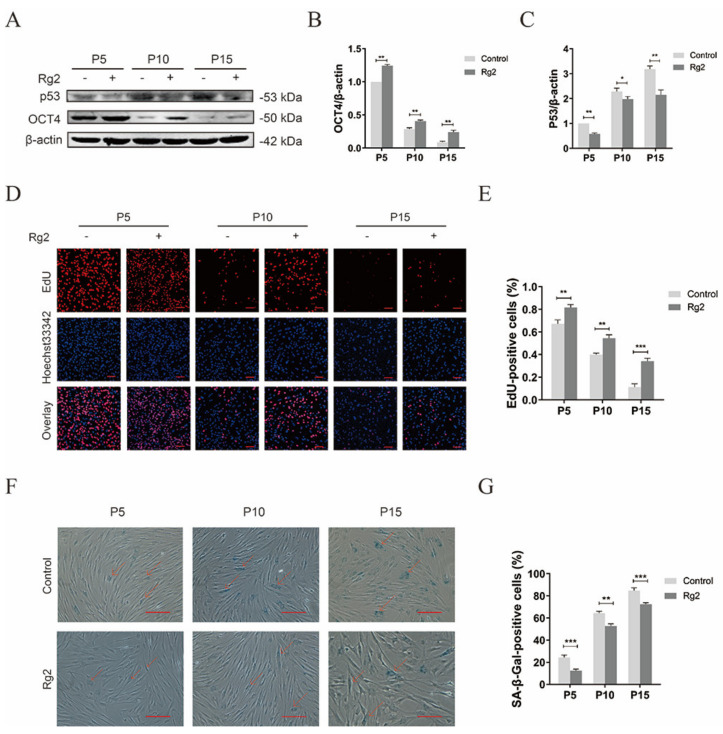
Ginsenoside Rg2 improved the longevity of pMSCs during long–term culture. (**A**) Western blot analysis with anti–*OCT4* and anti–P53 antibodies in pMSCs with/without Rg2 treatment at P5, P10, and P15. (**B**) Quantification of the *OCT4* expression levels in (**A**). (**C**) Quantification of the P53 expression levels in (**A**). (**D**) EdU staining of pMSCs with/without Rg2 treatment at P5, P10, and P15. Scale bar = 200 μm. (**E**) Quantification of the EdU staining in (**D**). (**F**) SA–β–gal staining of pMSCs with/without Rg2 treatment at P5, P10, and P15. SA–β–gal–positive cells were indicated by red arrows. Scale bar = 10 μm. (**G**) Quantification of the SA–β–gal staining in (**F**). * *p* < 0.05, ** *p* < 0.01, and *** *p* < 0.001; *n* = 3.

As Rg2 could maintain the stemness of pMSCs and stimulate proliferation, we next checked the effects of Rg2 on pMSCs during long-term culture. First, we checked the protein expression of *OCT4* and P53 in pMSCs at P5, P10, and P15 in the presence/absence of Rg2. The protein expression of *OCT4* significantly decreased in pMSCs at higher passage numbers, whether in Rg2-treated pMSCs or in cells without Rg2 (Figure 8A,B and Figure S5). However, higher *OCT4* protein expression was observed in Rg2-treated cells compared to the control group. Similarly, Rg2 treatment also resulted in low expression of P53 protein (Figure 8A,C and Figure S5). Consistent with these results, the percentage of EdU-positive cells remarkably decreased in pMSCs at higher passage numbers, whereas the administration of Rg2 upregulated a percentage of EdU-positive cells (Figure 8D,E). Meanwhile, we found that the administration of Rg2 downregulated the numbers of SA-β-gal-positive cells (Figure 8D,F). Taking together, long-term culture of pMSCs with Rg2 can help maintain stemness and promote proliferation, as well as inhibit aging.

## 4. Discussion

MSCs have the potential of self-renewal and multi-directional differentiation, including adipocytes and muscle cells [11,51,52,53,54], and thus are considered one of the most advantageous seed cells for cultured animal cell food [55]. However, the replicative senescence of porcine MSCs during in vitro expansion limits their application in the large-scale industrial production of cultured animal cell food [56]. Therefore, it is of great significance to explore an effective method to promote the proliferation and delay the senescence of porcine MSCs.

An increasing amount of evidence indicates that basal autophagy serves as a key mechanism to regulate the proliferation, differentiation, and stemness maintenance of adult stem cells, including MSCs [57,58], muscle stem cells (MuSCs) [29], and hematopoietic stem cells (HSCs) [59]. Human MSCs have been demonstrated to possess constitutive autophagic flux due to the observed LC3 conversion (LC3-I to LC3-II) [57,58]. Accumulation of cellular damage during senescence activates stem cell autophagic flux to remove toxic material and maintain their stemness. Emerging evidence has revealed that the activation of autophagy can eliminate ROS and oxidative proteins in aged MSCs, thus keeping their stemness and genomic integrity [28,60,61].

The role of autophagy in MSC aging seems puzzling due to some contrary reports. Zheng et al. observed the increased expression of autophagy-related protein, including LC3-II, ATG7, and ATG12, in aging rat MSCs, thus considering that autophagy is activated during cellular senescence [30]. However, the increase in LC3-II is the result of the combination of autophagosome formation and blockage of autophagic degradation. Thus, it is necessary to analyze autophagic flux by blocking autophagy with bafilomycin A1. Contrary to activated autophagy in aged MSCs [30], more studies support that autophagy activity is impaired during aging [31,32,33]. Compared with young BMMSCs, aged cells showed reduced expression of Atg7, Beclin1, and LC3II/I and the accumulation of P62, as well as fewer autophagosomes [32]. After chloroquine (CQ) treatment, young BMMSCs possessed more LC3 dots compared to aged cells, indicating that aged BMMSCs might be characterized by impaired autophagy [32]. In addition, autophagy markedly decreased in aged BMMSCs under normoxic and hypoxic conditions [31]. Here, we found that although the expression of LC3II increased in aged pMSCs compared to young counterparts, p62 proteins accumulated, suggesting the potential blockage of autophagic flux. Accordingly, the number of autophagosomes first increased and then decreased during pMSC senescence, confirmed by the addition of bafilomycin A1. Autophagic flux is significantly impaired due to the blockage of autophagic degradation in P15 pMSCs compared with P5 cells. These results indicate that in the early stage of senescence, pMSCs need to activate autophagy in response to oxidative stress and damaged proteins, while in the late stage of senescence, cells display a decline in autophagy function, thus leading to reduced clearance ability. In line with the impaired ROS clearance during senescence, increased ROS levels were observed in aged pMSCs. Our data indicate that the ability of aged pMSCs to remove toxic substrates might be defective.

The activation of autophagy could protect MSCs from oxidative stress, thus resisting aging and promoting proliferation. The autophagic agonist rapamycin has been reported to alleviate the senescent features of aged MSCs [32,62]. Hypoxia can promote the self-renewal and proliferation of MSCs by activating autophagy [63,64]. Contrarily, the inhibition of autophagy could promote aging in MSCs. The autophagic inhibitor 3-methyladenine (3-MA) aggravates the aging of MSCs [32,62]. It is reported that blocking autophagy with kynurenine accelerates senescence in mice BMMSCs via the aryl hydrocarbon receptor pathway [65]. 

Our previous study confirmed the positive effect of ginsenoside Rg2 on autophagy induction [41]. However, it is not clear whether Rg2 has a retarding effect on MSC aging. Here, we demonstrated that Rg2 promotes the proliferation of porcine MSCs and slows down senescence by activating autophagy. Similar to our results, a number of natural and synthetic compounds that can activate autophagy have been demonstrated to inhibit the senescence of MSCs and increase their proliferative potential [62,66,67]. Autophagy induced by curcumin protects canine BMMSCs against replicative senescence during in vitro expansion, defined by the increased colony-forming unit–fibroblastic (CFU-F) capacity and decreased SA-β-gal activities [62]. A combination of 5-aminoimidazole-4-carboxamide ribonucleotide (AICAR), an AMPK activator, and nicotinamide (NAM), an activator of sirtuin1 (SIRT1), showed the protective effect of anti-senescence and proliferation promotion in MSCs [61]. Camphorquinone [67] and licochalcone D [66] have been reported to be able to activate autophagy via the adenosine-monophosphate-activated protein kinase (AMPK) signal pathway, therefore alleviating the H_2_O_2_-induced senescence of human BMMSCs in vitro and also inhibiting D-gal-induced aging in mice in vivo. 

Reactive oxygen species are known to be important risk factors affecting the aging of mesenchymal stem cells [68,69,70]. It is known that ROS production increases with age, leading to oxidative DNA damage and decreased proliferation of stem cells [71]. D-gal induced accelerated senescence has been used as a conventional experimental model to study cell senescence [72,73]. Previous studies have also shown that D-gal can significantly induce senescence in MSCs by promoting ROS production [74]. Here, we also found that the percentage of SA-β-gal-positive cells significantly increased and the number of EdU-positive cells remarkably decreased in D-gal-treated pMSCs, whereas the changes were reversed with Rg2. Rg2 treatment also inhibited D-gal-induced upregulation of P53 expression and downregulation of *OCT4* expression, suggesting that Rg2 prevents D-gal-mediated senescence in porcine MSCs. A recent report showed that Rg2 could delay D-gal-induced brain aging and recover impaired memory function in mice by increasing mitochondrial autophagy flux and relieving oxidative stress [42]. Similarly, ginsenoside Rg1 has been shown to have protective effects in multiple tissues of mice with D-gal-induced aging through attenuating oxidative stress [75,76,77]. Upregulating autophagy with by rapamycin has been shown to inhibit ROS generation and attenuate senescence caused by D-gal in rat BMMSCs [78]. Furthermore, our data demonstrated that Rg2 can protect porcine MSCs against the oxidative stress signal triggered by D-gal, as evidenced by the enhanced SOD activity and reduced MDA and ROS levels. This result was coincident with a previous finding that Rg2 effectively inhibits oleic acid and palmitic acid (OA&PA)-induced ROS generation in mouse primary hepatocytes [79]. The combined treatment of Rg2 and Rh1 has been found to significantly suppress LPS-induced excessive ROS accumulation in HepG2 cells [38].

One of the major regulators of autophagy is the adenosine-monophosphate-activated protein kinase (AMPK) signaling pathway [80]. AMPK can inhibit the activation of mammalian target of rapamycin (mTOR) through phosphorylating raptor, while mTOR functions as a critical negative regulator of autophagy by inhibiting Unc-51-like kinase 1 (ULK1) activation [81,82]. In addition, AMPK can trigger autophagy by directly phosphorylating ULK1 at multiple sites, such as S317, S467, and S777. [83,84]. The AMPK-mediated activation of autophagy has been reported to ameliorate D-gal-induced senescence in multiple tissues, including the heart [66,67], hippocampus [66,85,86], kidney [87], and skeletal muscle [88]. In human BMMSCs, licochalcone D or camphorquinone can induce autophagy and reduce H_2_O_2_-induced senescence via the AMPK signal pathway [66,67]. Rg2 has been reported to activate the AMPK signal in multiple cell lines, including 3T3-L1 preadipocytes [89], HepG2 cells [90], MCF-7 cells [39], Neuro2A cells [41], and PC12 cells [41]. Similarly, our data confirmed that pre-incubation with Rg2 significantly upregulates LC3-II expression and activates authophagy in D-gal-treated pMSCs via the AMPK signaling pathway.

In addition, we found that 100 μM of Rg2 can significantly enhance the proliferative capacity of porcine MSCs and inhibit replicative senescence during long-term culture in vitro. A recent study focused on the positive effect of Rg2 on the proliferation of induced-pluripotent-stem-cell-derived endothelial cells (iPSC-ECs) for clinical application [91]. Similar to the concentration of Rg2 used in our study, 10–200 μM of Rg2 was found to remarkably upregulate the EdU-positive cellular number of iPSC-ECs after three passages [91]. Mechanically, the stimulatory effect of Rg2 on iPSC-EC proliferation depends on mTOR-independent AMPK/ULK1-mediated autophagy. Furthermore, two recent studies on the use of Rg2 in the development of functional foods reported that the working concentration of Rg2 in the cells is approximately 80 μM [35,89], which is similar to the concentration of Rg2 (25–100 μM) used in our study.

## 5. Conclusions

Taken together, our findings suggest that in the early stage of senescence, pMSCs enhance autophagosome formation in respond to oxidative stress, while in the late stage, aged cells display impaired autophagic flux, thus leading to reduced clearance ability. Furthermore, ginsenoside Rg2 improves the longevity of porcine MSCs by inducing AMPK-mediated protective autophagy. Ginsenoside Rg2 may be an effective protector of MSC senescence induced by oxidative stress. These findings highlight the positive role of Rg2 in porcine MSC expansion in vitro.

## Figures and Tables

**Table 1:** The primer sequences in this study.

Gene	Primer Sequence
*OCT4*	F:5′-GTCGCCAGAAGGGCAAAC-3′
	R:5′-CAGGGTGGTGAAGTGAGGG-3′
*KI67*	F:5′-TTCATTCACTGGTCCTCG-3′
	R:5′-TTAGCCACTTCTGACTTTC-3′
*GAPDH*	F:5′-TGAAGGTCGGAGTGAACG-3′
	R:5′-CGTGGGTGGAATCATACTGG-3′

## Data Availability

The data presented in this study are available on request from the corresponding author.

## Supplementary Materials

The following supporting information can be downloaded at: https://www.mdpi.com/article/10.3390/foods12051075/s1, Figure S1: Original Images of figure 2 for Blots; Figure S2: Original Images of figure 3 for Blots; Figure S3: Original Images of figure 5 for Blots; Figure S4: Original Images of figure 7 for Blots; Figure S5: Original Images of figure 8 for Blots.
