# Supplementary material for: Ginsenoside Rg2 Promotes the Proliferation and Stemness Maintenance of Porcine Mesenchymal Stem Cells through Autophagy Induction
Source: Foods. 2023 Mar 2;12(5):1075. doi: 10.3390/foods12051075 (PMC10000966; doi:10.3390/foods12051075)
Supplement: Supplementary file 1 [file foods-12-01075-s001.zip › foods-2172057-supplementary.pdf]

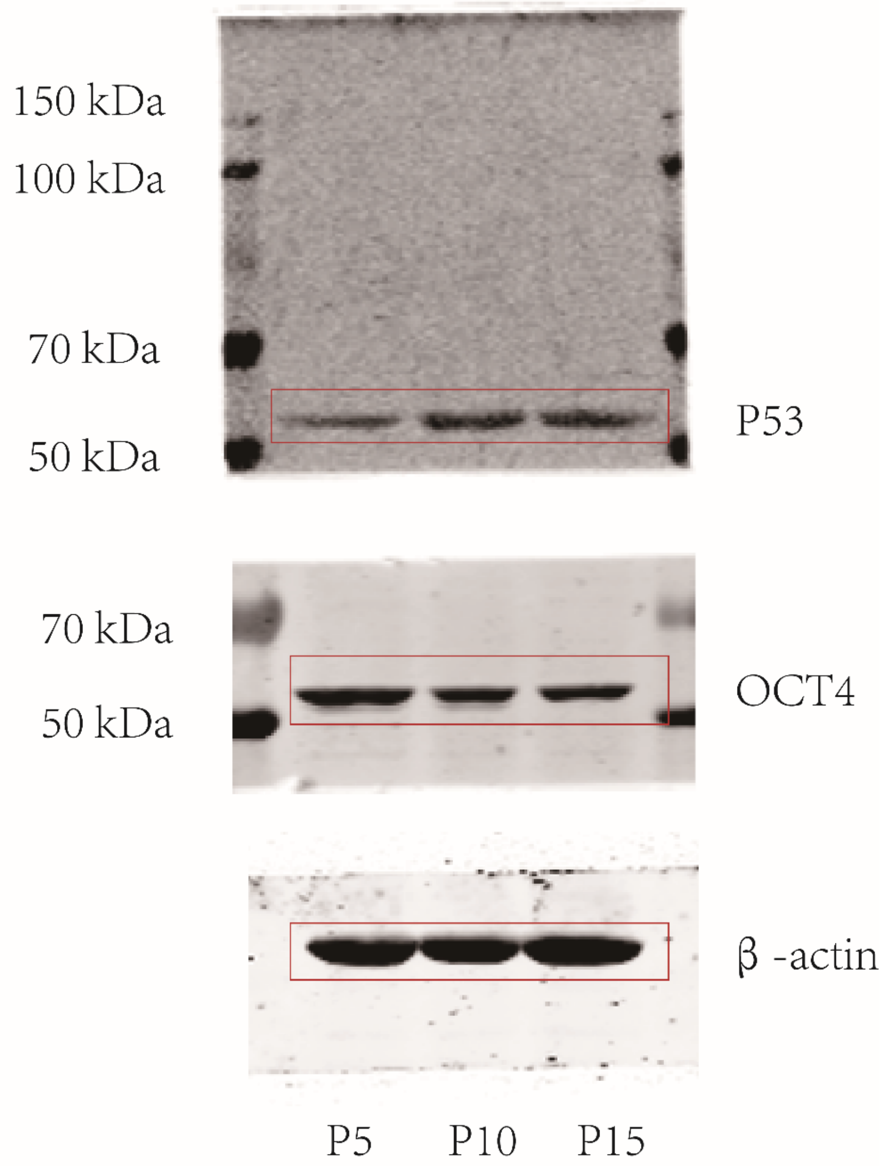

Figure S1. Original Images of figure 2 for Blots.

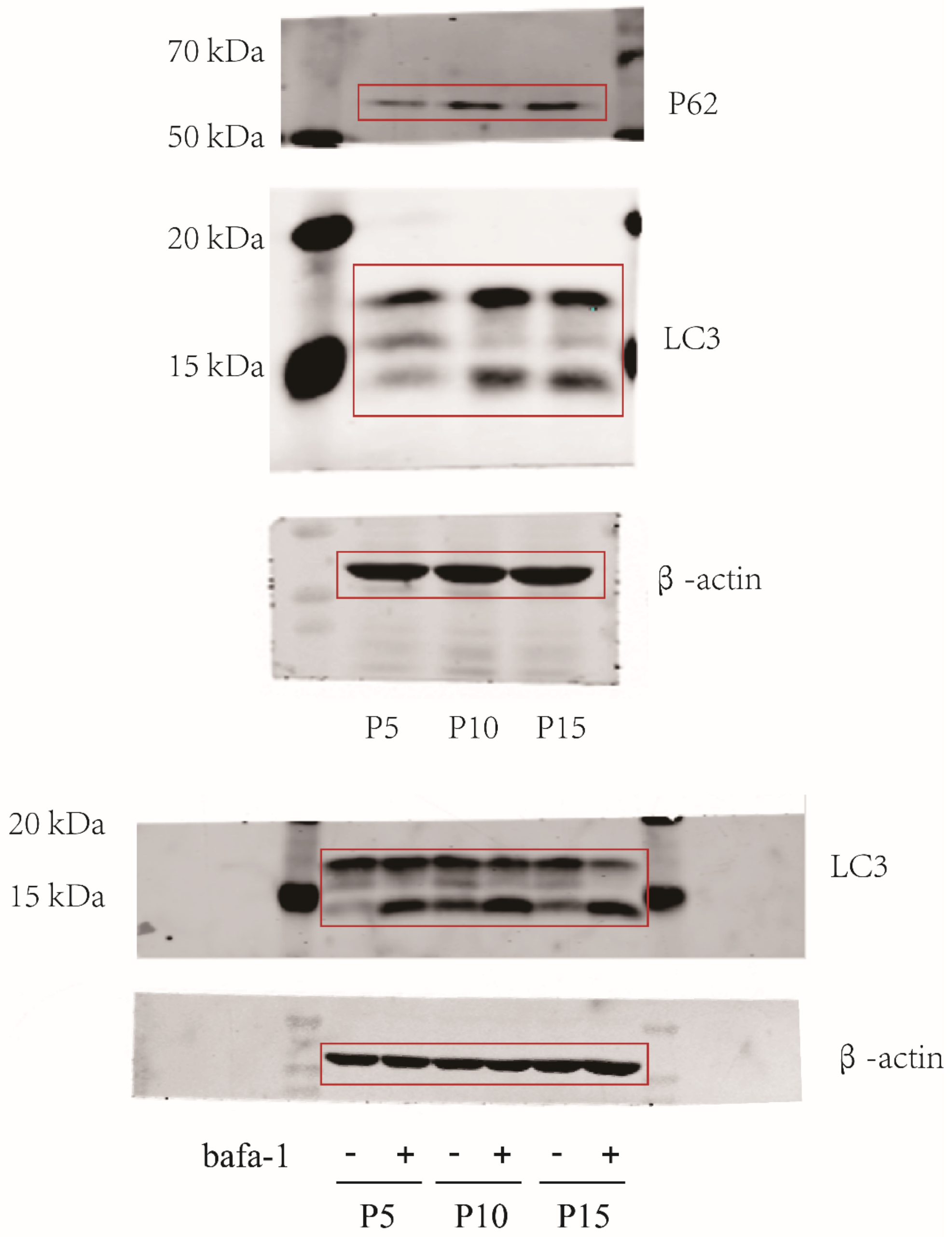

Figure S2. Original Images of figure 3 for Blots.

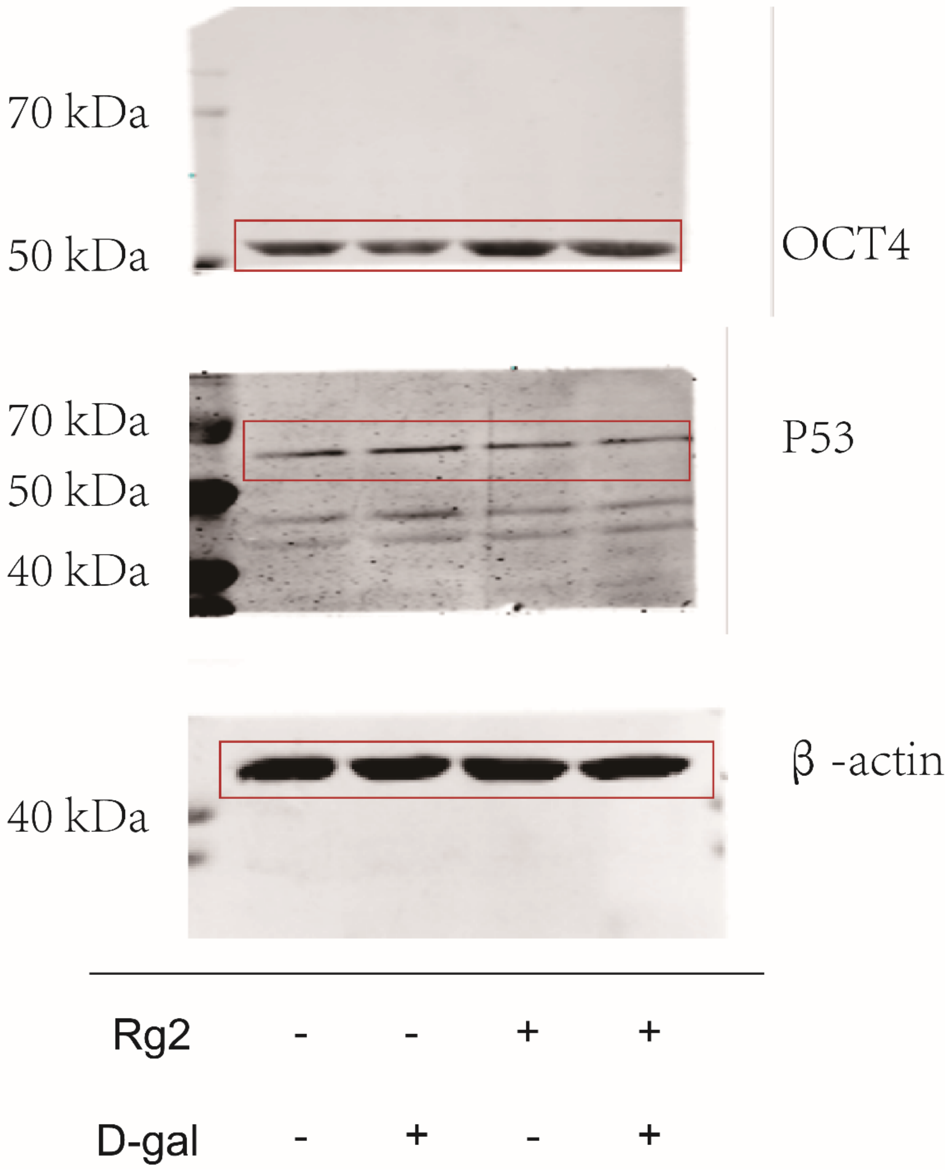

Figure S3. Original Images of figure 5 for Blots.

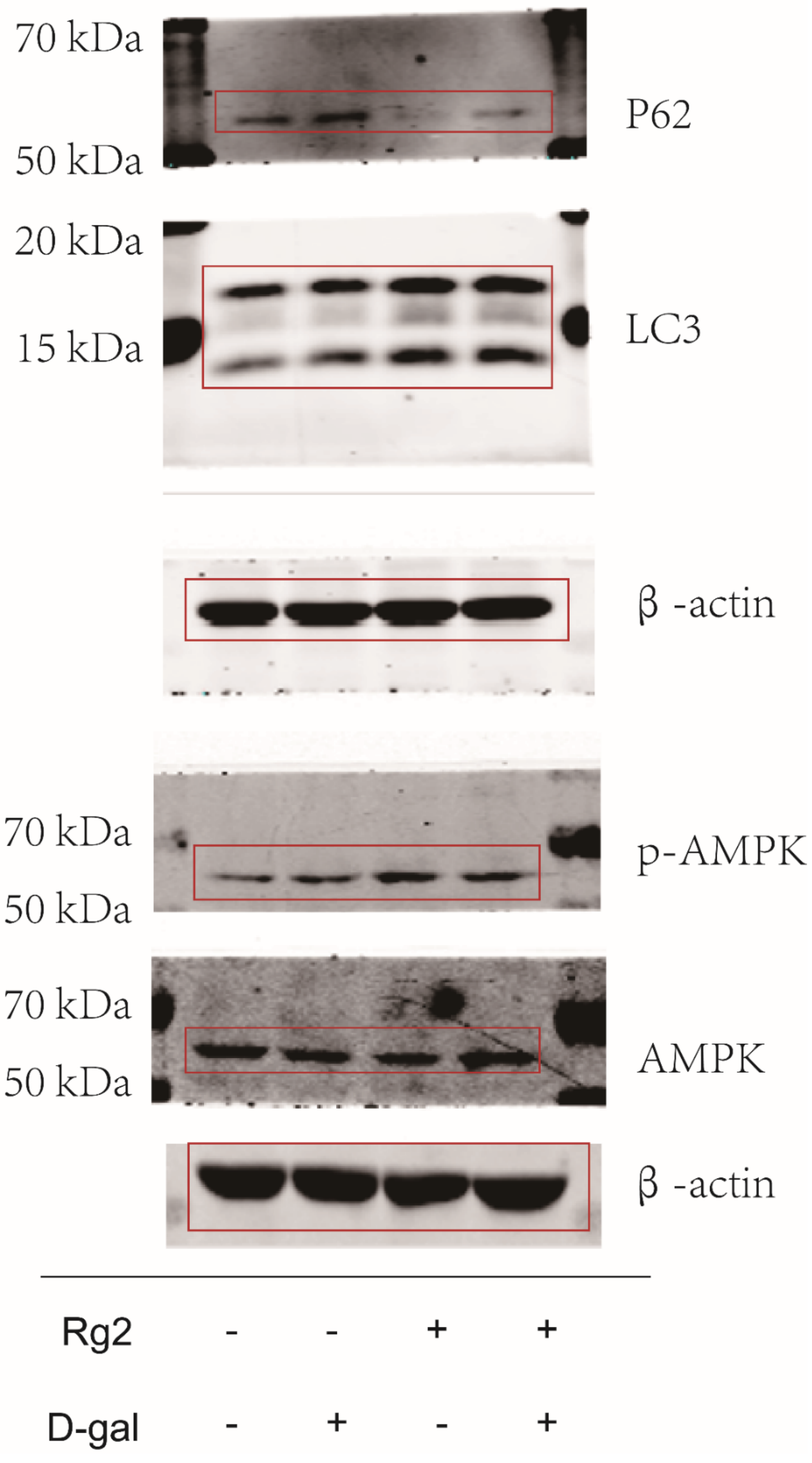

Figure S4. Original Images of figure 7 for Blots.

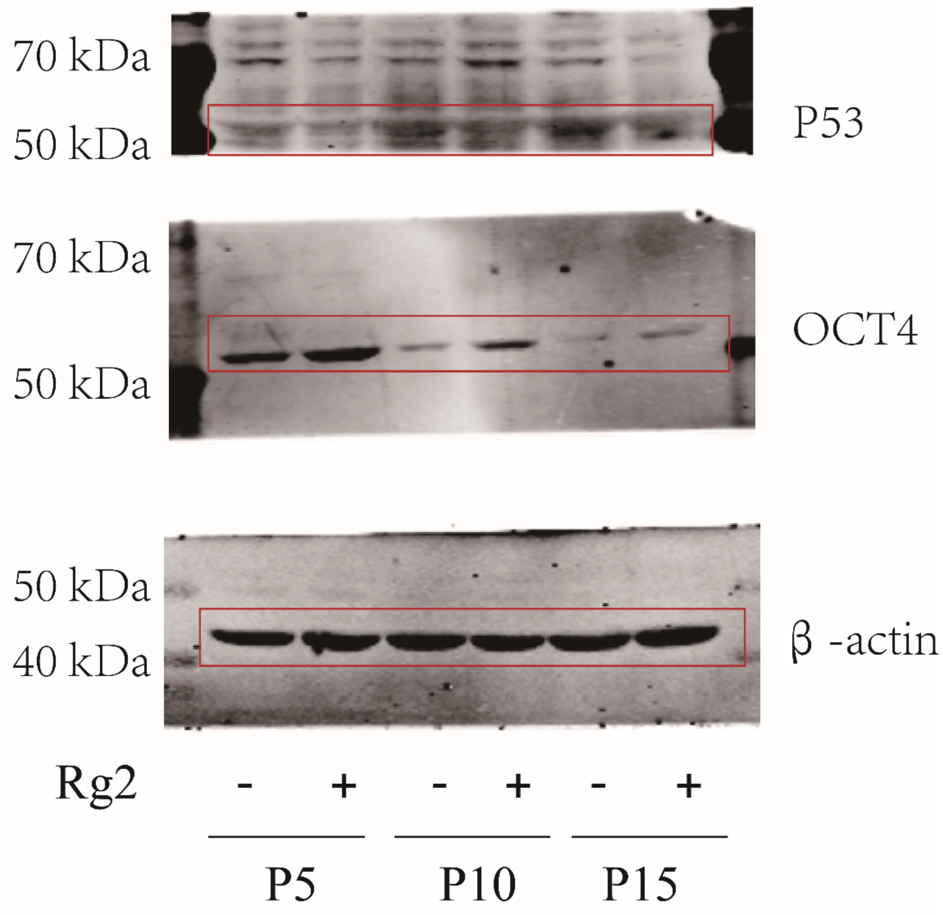

Figure S5. Original Images of figure 8 for Blots.
